# Strategic Drivers Behind Early Withdrawal of Orphan Designations in the EU: A Retrospective Analysis (2000–2024)

**DOI:** 10.1007/s43441-025-00890-z

**Published:** 2025-10-23

**Authors:** Luísa Bouwman, Micael Castanheira, Georges Siotis

**Affiliations:** 1 Pharmacist specialist in Regulatory Affairs, Utrecht, The Netherlands; 2 Fonds National de la Recherche Scientifique (FNRS) and European Centre for Advanced Research in Economics and Statistics (ECARES), Université Libre de Bruxelles, Brussels, Belgium; 3 Economics Department, University Carlos III de Madrid, Madrid, Spain; 4 Centre for Economic Policy Research (CEPR), Paris, France; 5 Interdisciplinary Institute for Innovation in Healthcare (i3h), Université Libre de Bruxelles, Brussels, Belgium

**Keywords:** Orphan regulation, Orphan designations, Incentives, Orphan medicines

## Abstract

**Purpose:**

The orphan legislation came into force in the European Union (EU) in 2000, providing incentives for the development of orphan medicines. To be eligible for incentives, the applicant needs to apply for an orphan designation (OD). However, at any time, the marketing authorisation holder (MAH) can request the removal of the OD. The possible motives underpinning premature removal of OD have been the subject of speculation. Our aim is to study every early OD removal for the orphan medicinal products (OMPs) approved in the EU between 2000 and 2024 and determine the main reasons behind this phenomenon.

**Methods:**

We identified all the orphan medicines approved between 2000 and 2024. We considered approval date by the European Commission (EC), orphan designation (OD) status, company name, active substance name, trade name, ATC code and Therapeutic Area, and the date of the removal of the OD. Information on the OD withdrawal was cross-checked with the documents on the EMA website, and the legal status of the patent and supplementary protection certificates (SPC) was checked at the European Patent Register.

**Results:**

During the period 2000–2024, 285 OMPs were approved by the EC. Overall, 41 (11.8%) orphan designations were prematurely removed, corresponding to 23 different OMPs.

**Conclusions:**

Three main motives for the early removal of the OD were identified: lack of clinical evidence supporting the significant benefit for the new indication proposed, the companies’ preference towards SPC extensions for the paediatric indication (instead of the two additional years of marketing exclusivity), or the new therapeutic indication added is not rare. There is no evidence of commercial “pay to enter” agreements between pharmaceutical companies.

## Introduction

The orphan legislation came into force in the European Union (EU) in 2000, with the promulgation of Regulation (EC) No 141/2000, addressing the need to offer incentives for the development and marketing of medicines for rare diseases [1]. The EU Orphan Regulation establishes that a medical product can be designated as orphan medicine if: (a) it is intended for the diagnosis, prevention or treatment of a life-threatening or chronically debilitating condition affecting no more than 5 in 10,000 persons in the European Union at the time of submission of the designation application (prevalence criterion), (b) it is intended for the diagnosis, prevention or treatment of a life-threatening, seriously debilitating or serious and chronic condition and that, without incentives, it is unlikely that expected sales of the medicinal product would cover the investment in its development, and (c) no satisfactory method of diagnosis, prevention or treatment of the condition concerned is authorized, or, if such method exists, the medicinal product will be of significant benefit to those affected by the condition.

The incentives created by the Orphan Regulation include [2]: access to protocol assistance (a form of scientific advice provided by the European Medicines Agency (EMA) specifically for orphan medicines), fee reductions (with additional reductions to small and medium enterprises), access to EU-funded research (and country-level incentives), and market exclusivity of 10 years (+ 2 if paediatric indication is approved). This market exclusivity period is a market-level protection against the competition of similar medicines for the same indication. Market exclusivity differs from Intellectual Property Rights (IPRs): the latter grants the holder monopoly rights on the active pharmaceutical ingredient (API) and the associated processes. In contrast, market exclusivity provides for exclusivity on the therapeutic indication. Hence, under standard conditions, the MAH of an OMP is protected from entry by competing OMPs (e.g., “me too” products).

To be eligible for incentives, the applicant must apply for one or more orphan designations through an EU procedure. The Committee for Orphan Medicinal Products (COMP) will assess the application and adopt an opinion within 90 days. It forwards this to the European Commission (EC) for adoption of a decision [3–5]. However, at any time, the marketing authorisation holder can submit a request to withdraw the orphan designation.

The removal of an orphan designation is in accordance with Article 5(12) of the Orphan Regulation and is irreversible [1]. Once all of the orphan designations associated with an approved medicine have expired or have been removed by the sponsor, the medicine ceases to be classified as an orphan medicine and no longer benefits from the orphan incentives. In 2021, Montanaro et al. [6]. published a study addressing the intriguing question related to the “voluntary” withdrawal of orphan designations by the marketing authorisation holder (MAH) before the end of market exclusivity. These authors speculate that one plausible motive for the premature withdrawals could be commercial “pay-to-enter agreements” between pharmaceutical companies. Under that scenario, the economic logic underpinning the voluntary removal of the OD status would be to allow a competitor to enter the market with a similar OMP and hence benefit from 10 years of market exclusivity. For this to be mutually beneficial (“incentive compatible” in economics’ jargon), the entrant would have to compensate the MAH financially. The economic logic underpinning a hypothetical “pay to enter agreement” is similar to that of a “pay for delay”. In both cases, a payment is made to maintain some form of exclusivity beyond the initial end date. While the economic incentives are similar, the legal status differs: “pay to enter” is presumably legal, while “pay for delay” is not [7, 8].

Our study aims to accurately trace back the reasons for each premature removal of an orphan designation in the EU. We identified every orphan medicinal product that the EC approved between 2000 and 2024 and for which the orphan designation has been withdrawn “upon request of the MAH.”

To our best knowledge, no other research has been conducted to clear the fog around this phenomenon, whether in the EU or in the United States. We believe that this finding is relevant for the ongoing discussion on the upcoming revised General Pharmaceutical Legislation (GPL), which could be the most significant reform in the last 30 years [9].

## Methods

The publicly available search-database of the EMA [10] was used to identify all orphan medicines approved in EU through the centralised procedure between 1st January 2000 and 31st December 2024, including those that were subsequently withdrawn. We included all products with orphan designation (OD) at the time of Committee for Human Medicinal Products (CHMP) opinion. The following data were extracted from the EMA search-data base: date of approval, orphan designation number, description and status (active, withdrawn or expired), marketing authorisation holder’s company name, active substance name, trade name, Anatomical Therapeutical Chemical (ATC) code and Therapeutic Area. The date of the removal of the OD was extracted, if earlier than the end of the market exclusivity period. Only publicly available documentation has been used.

The orphan maintenance assessment reports (OMARs) available at the EMA’s website were analysed (if available) to check the outcome of the review of criteria for orphan designation and the COMP list of issues (existing methods, significant benefit, number of people affected or at risk, chronically debilitating and/or life-threatening nature). For the cases where the OMAR is not available (i.e., prior to 2018), the document with procedural steps taken and scientific information after authorisation, also available on the EMA’s website, was checked to identify any possible change to the therapeutic indication approved in the same year of the OD removal. For the remaining cases, which could not be explained by changes to the therapeutic indication, patent-related reasons were explored. The legal status of the patent and supplementary protection certificates (SPC) was checked at the European Patent Register (9). The documentation related to Paediatric Investigation Plans (PIPs) was also analysed. The possible reason(s) for the early removal of the OD was annotated. The data extracted were used to build a database containing all OMPs approved by the EC between 2000 and 2024 and for which the OD was prematurely removed (i.e., before the end of the market exclusivity period). Descriptive statistical analysis was applied.

## Results

In the period under study, 285 OMPs were approved by the EC, corresponding to 347 ODs. From these, 176 (50.7%) ODs were still valid, 87 (25.1%) OD have expired, and 84 (24.2%) were withdrawn upon request of the MAH (*cutoff date: 31st December 2024*).

**Fig. 1:**
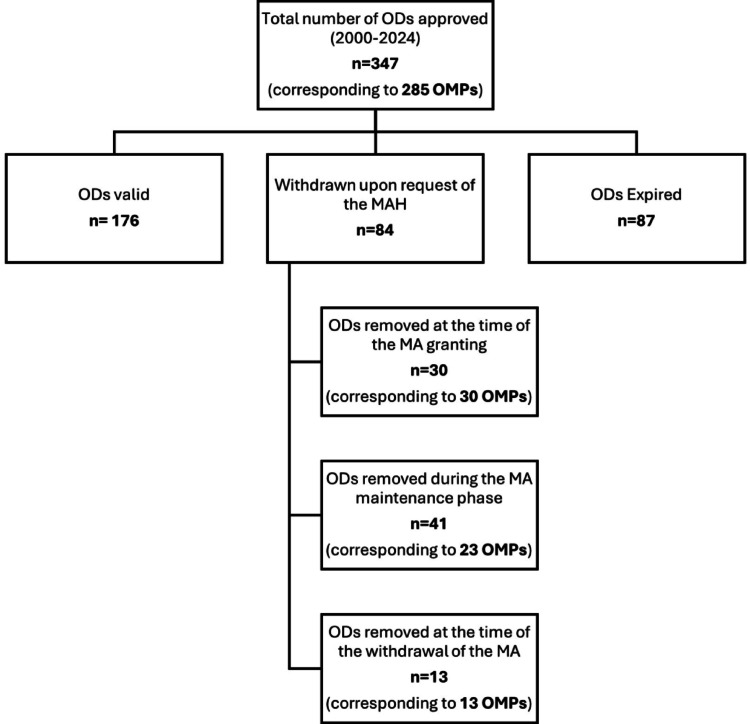
Results in terms of number of orphan designations approved between 2000 and 2024 and their current status (*data lock point: 31st December 2024*).

In the group of the ODs removed upon request of the MAH (*n* = 84) it is possible to distinguish three different situations (Fig. 1): 30 (35.3%) ODs were removed at the time of granting the MA, 13 (15.3%) were removed at the time of the withdrawal of the Orphan MA by the EC and 41 (48.8%) ODs were removed during maintenance phase, before the end of the market exclusivity period.

Table 1 lists the 41 ODs that were removed upon request of the MAH during the maintenance phase and before the end of the market exclusivity period. These correspond to 23 different OMPs, for which the median time between the marketing authorization (MA) and the withdrawal of the OD was 3 years (with a minimum of 1 year and a maximum of 11 years).

**Table 1:** Orphan designations withdrawn from the Community Register of orphan medicinal products from January 2000 to December 2024

EU OD number	Year of MA approval by the EC	Type of MA granted	MAH	Active substance	Tradename	ATC code	Therapeutic area	OD description	Status of the OD (withdrawal date)	Years from MA to OD removal	(Likely) Reason for the OD removal
EU/3/08/595	2012	Conditional approval	Takeda	brentuximab vedotin	Adcetris®	L01XC12	Lymphoma, Non-Hodgkin; Hodgkin Disease	Treatment of peripheral T-cell lymphoma	Withdrawn (June 2024)	12	Unclear. Takeda submitted end 2023 type II variation to extend indication. The overall B/R was considered negative. Takeda decided to withdraw the application in Feb 2024 (withdrawal letter variation II/109).
EU/3/08/596	2012	Conditional approval	Takeda	brentuximab vedotin	Adcetris®	L01XC12	Lymphoma, Non-Hodgkin; Hodgkin Disease	Treatment of Hodgkin lymphoma	Withdrawn (June 2024)	12	Unclear. Takeda submitted end 2023 type II variation to extend indication. The overall B/R was considered negative. Takeda decided to withdraw the application in Feb 2024 (withdrawal letter variation II/109).
EU/3/11/939	2017	Conditional approval	Takeda	brentuximab vedotin	Adcetris®	L01XC12	Lymphoma, Non-Hodgkin; Hodgkin Disease	Treatment of cutaneous T-cell lymphoma	Withdrawn (June 2024)	7	Unclear. Takeda submitted end 2023 type II variation to extend indication. The overall B/R was considered negative. Takeda decided to withdraw the application in Feb 2024 (withdrawal letter variation II/109).
EU/3/07/518	2014	Standard	Bayer	riociguat	Adempas®	C02KX05	Hypertension, Pulmonary	Treatment of pulmonary arterial hypertension	Withdrawn (2022)	8	PIP completed in 2022. SPC extension preferred (EP1506193).
EU/3/07/449	2009	Standard	Novartis	everolimus	Afinitor®	L01XE10	Carcinoma, Renal Cell	Treatment of renal cell carcinoma	Withdrawn (2011)	2	Extension of indication to include treatment of advanced gastro-entero-pancreatic neuroendocrine tumours. This indication does not fall within any orphan designation.
EU/3/08/581	2010	Conditional approval	GlaxoSmithKline	ofatumumab	Arzerra®	L01XC10	Leukemia, Lymphocytic, Chronic, B-Cell	Treatment of chronic lymphocytic leukaemia	Withdrawn (Dec 2018)	8	Withdrawal of the MA for commercial reasons in Feb 2019. Therefore, OD was also removed.
EU/3/15/1590	2017	Conditional approval	Merck Group	avelumab	Bavencio®	L01FF04	Neuroendocrine Tumors	Treatment of Merkel cell carcinoma	Withdrawn (2019)	2	The new indication, renal carcinoma, does not fall within any orphan designation.
EU/3/10/762	2013	Standard	Pfizer	bosutinib	Bosulif®	L01EA04	Leukemia, Myeloid	Treatment of Chronic Myeloid Leukemia. Extension of indication treatment of adult patients with newly diagnosed Philadelphia Chromosome positive (Ph+) Chronic Phase (CP) Chronic Myelogenous Leukaemia (CML) approved March 2018.	Withdrawn (2018)	5	Withdrawn in March 2018 upon MAH request at the time of granting of a change to the terms of the MA. Significant benefit not proven (OMAR, 2018).
EU/3/12/1004	2014	Standard	Eli Lilly	ramucirumab	Cyramza®	L01XC	Stomach Neoplasms	Treatment of gastric cancer	Withdrawn (2015)	1	The new indication, colorectal cancer, submitted in 2015, does not fall within any orphan condition.
EU/3/19/2145	2021	Standard	Roche	risdiplam	Evrysdi®	M09AX10	Muscular Atrophy, Spinal	Treatment of spinal muscular atrophy	Withdrawn (2023)	2	In 2023 an extension of indication to include treatment of patients below 2 months of age was approved. At that time MAH requested the withdrawal of the OD. SPC extension preferred. Request not made yet, but the MAH still has time to request it (EP3143025).
EU/3/01/061	2001	Standard	Novartis	imatinib	Glivec®	L01EA01	Myelodysplastic-Myeloproliferative Diseases	Treatment of malignant gastrointestinal stromal tumours	Withdrawn (2012)	10	PIP results available in 2012. SPC extension preferred (EP0564409).
EU/3/05/305	2006	Standard	Novartis	imatinib	Glivec®	L01EA01	Dermatofibrosarcoma	Treatment of dermatofibrosarcoma protuberans	Withdrawn (2012)	6	PIP results available in 2012. SPC extension preferred (EP0564409).
EU/3/05/304	2006	Standard	Novartis	imatinib	Glivec®	L01EA01	Precursor Cell Lymphoblastic Leukemia-Lymphoma	Treatment of acute lymphoblastic leukaemia	Withdrawn (2012)	6	PIP results available in 2012. SPC extension preferred (EP0564409).
EU/3/05/320	2006	Standard	Novartis	imatinib	Glivec®	L01EA01	Hypereosinophilic Syndrome	Treatment of chronic eosinophilic leukaemia and the hypereosinophilic syndrome	Withdrawn (2012)	6	PIP results available in 2012. SPC extension preferred (EP0564409).
EU/3/05/340	2006	Standard	Novartis	imatinib	Glivec®	L01EA01	Myelodysplastic-Myeloproliferative Diseases	Treatment of myelodysplastic / myeloproliferative diseases	Withdrawn (2012)	6	PIP results available in 2012. SPC extension preferred (EP0564409).
EU/3/07/439	2009	Exceptional Circumstances	Novartis	canakinumab	Ilaris®	L04AC08	Cryopyrin-Associated Periodic Syndromes; Arthritis, Juvenile Rheumatoid; Arthritis, Gouty	Treatment of cryopirin-associated periodic syndromes	Withdrawn (2010)	1	The new indication, treatment of gouty arthritis, does not fall within any orphan condition.
EU/3/12/984	2014	Standard	Janssen-Cilag	ibrutinib	Imbruvica®	L01EL01	Leukemia, Lymphocytic, Chronic	Treatment of chronic lymphocytic leukaemia	Withdrawn (2021)	7	SPC extension preferred (EP2201840).Request not made yet but the MAH still has time to do it.
EU/3/13/1115	2014	Standard	Janssen-Cilag	ibrutinib	Imbruvica®	L01EL01	Lymphoma, Mantle-Cell;	Treatment of mantle-cell lymphoma	Withdrawn (2021)	7	SPC extension preferred (EP2201840).Request not made yet but the MAH still has time to do it.
EU/3/14/1264	2014	Standard	Janssen-Cilag	ibrutinib	Imbruvica®	L01EL01	Leukemia, Lymphocytic, Chronic, B-Cell	Treatment of lymphoplasmacytic lymphoma	Withdrawn (2021)	7	SPC extension preferred (EP2201840).Request not made yet but the MAH still has time to do it.
EU/3/08/572	2012	Standard	Novartis	ruxolitinib	Jakavi®	L01EJ01	Myeloproliferative Disorders	Treatment of chronic idiopathic myelofibrosis	Withdrawn (2015)	3	Re-assessment of orphan Criteria at the time of type II variation to add new indication. Significant benefit not proven.
EU/3/09/620	2012	Standard	Novartis	ruxolitinib	Jakavi®	L01EJ01	Myeloproliferative Disorders	Treatment of myelofibrosis secondary to polycythaemia vera or essential thrombocythaemia	Withdrawn (2015)	3	Re-assessment of orphan Criteria at the time of type II variation to add new indication. Significant benefit not proven.
EU/3/14/1244	2015	Standard	Novartis	ruxolitinib	Jakavi®	L01EJ01	Polycythemia vera	Treatment of polycythaemia vera -	Withdrawn (2015)	0	Re-assessment of orphan Criteria at the time of type II variation to add new indication. Significant benefit not proven.
EU/3/13/1121	2015	Standard	Eisai Europe	lenvatinib	Lenvima®	L01XE29	Thyroid Neoplasms	Treatment of papillary thyroid cancer	Withdrawn (2018)	3	Lack of clinical data to support clinical benefit is only applicable to hepatocellular carcinoma. However, the same MA cannot include orphan and non orphan indications.
EU/3/13/1119	2015	Standard	Eisai Europe	lenvatinib	Lenvima®	L01XE29	Thyroid Neoplasms	Treatment of follicular thyroid cancer	Withdrawn (2018)	3	Lack of clinical data to support clinical benefit is only applicable to hepatocellular carcinoma. However, the same MA cannot include orphan and non orphan indications.
EU/3/15/1460	2018	Standard	Eisai Europe	lenvatinib	Lenvima®	L01XE29		Treatment of hepatocellular carcinoma	Withdrawn (2018)	0	Significant benefit not proven (OMAR 2018).
EU/3/07/501	2014	Standard	AstraZeneca	olaparib	Lynparza®	L01XK01	Ovarian Neoplasms	Treatment of ovarian cancer	Withdrawn (2018)	4	Re-assessment of orphan criteria at the time of type II variation: significant benefit not proven (OMAR, 2018).
EU/3/13/1123	2018	Standard	Boehringer Ingelheim	nintedanib	Ofev®	L01EX09	Idiopathic pulmonary fibrosis	Treatment of idiopathic pulmonary fibrosis	Withdrawn (2020)	5	PIP completed. Patent extension preferred (EP1224170).
EU/3/16/1724	2020	Standard	Boehringer Ingelheim	nintedanib	Ofev®	L01EX09	Systemic sclerosis	Treatment of systemic sclerosis (new indication)	Withdrawn (2020)	0	At the time of granting of the new indication the COMP recomended keeping the OD. However, MAH preferred SPC extension (EP1224170).
EU/3/11/909	2013	Standard	Janssen-Cilag	macitentam	Opsumit®	C02KX04	Hypertension, Pulmonary	Treatment of pulmonary hypertension	Withdrawn (2023)	10	Pediatric data available. MAH preferred SPC extension (EP1345920).
EU/3/04/192	2012	Standard	Bristol-Myers Squibb (->Celgene)	lenalidomide	Revlimid®	L04AX04	Multiple Myeloma	New indication: treatment of patients with transfusion dependent anaemia due to low- or intermediate-1-risk myelodysplastic syndromes	Withdrawn (2019)	7	All ODs withdrawn in 2019: re-assessment at the time of type II variation to add new indication (treatment of follicular lymphoma). Significant benefit not proven (OMAR, 2019).
EU/3/11/924	2015	Standard	Bristol-Myers Squibb (->Celgene)	lenalidomide	Revlimid®	L04AX04	Lymphoma, Mantle-Cell	New indication: treatment of adult patients with previously untreated multiple myeloma who are not eligible for transplant	Withdrawn (2019)	4	All ODs withdrawn in 2019: re-assessment at the time of type II variation to add new indication (treatment of follicular lymphoma). Significant benefit not proven (OMAR, 2019).
EU/3/11/924	2016	Standard	Bristol-Myers Squibb (->Celgene)	lenalidomide	Revlimid®	L04AX04	Lymphoma, Mantle-Cell	New indication: Treatment of mantle cell lymphoma	Withdrawn (2019)	3	All ODs withdrawn in 2019: re-assessment at the time of type II variation to add new indication (treatment of follicular lymphoma). Significant benefit not proven (OMAR, 2019).
EU/3/04/192	2017	Standard	Bristol-Myers Squibb (->Celgene)	lenalidomide	Revlimid®	L04AX04	Multiple Myeloma	New indication: Treatment of adult patients with newly diagnosed multiple myeloma who have undergone autologous stem cell transplantation	Withdrawn (2019)	2	All ODs withdrawn in 2019: re-assessment at the time of type II variation to add new indication (treatment of follicular lymphoma). Significant benefit not proven (OMAR, 2019).
EU/3/12/1097	2019	Standard	Bristol-Myers Squibb (->Celgene)	lenalidomide	Revlimid®	L04AX04	Follicular lymphoma	New indication: Treatment of follicular lymphoma	Withdrawn (2019)	0	Re-assessment of orphan criteria at the time of type II variation: significant benefit not proven (OMAR, 2019).
EU/3/04/192	2019	Standard	Bristol-Myers Squibb (->Celgene)	lenalidomide	Revlimid®	L04AX04	Multiple Myeloma	New indication: treatment combination with bortezomib and dexamethasone of adult patients with previously untreated multiple myeloma who are not eligible for transplant	Withdrawn (2019)	0	All ODs withdrawn in 2019: re-assessment at the time of type II variation to add new indication (treatment of follicular lymphoma). Significant benefit not proven (OMAR, 2019).
EU/3/07/467	2010	Standard	GlaxoSmithKline	eltrombopag	Revolade®	B02BX05	Purpura, Thrombocytopenic, Idiopathic	Treatment of idiopathic thrombocytopenic purpura	Withdrawn (2012)	2	Orphan designation withdrawn at the time of type II variation to extend the indication (treatment of HCV associated thrombocytopenia). Significant benefit not proven.
EU/3/05/268	2006	Standard	Pfizer	sunitinib	Sutent®	L01EX01	Carcinoma, Renal Cell	Treatment of renal cell carcinoma	Withdrawn (2008)	2	Re-assessment of orphan criteria at the time of type II variation: significant benefit not proven.
EU/3/05/267	2006	Standard	Pfizer	sunitinib	Sutent®	L01EX01	Gastrointestinal Stromal Tumors	Treatment of malignant gastrointestinal stromal tumours	Withdrawn (2008)	2	As a consequence of the lost of ODD for renal cell carcinoma, the ODD for the other approved indication has also to be removed since it is not possible to keep in the same MA orphan and non-orphan indications.
EU/3/22/2581	2023	Conditional approval	AbbVie	epcoritamab	Tepkinly®	L01FX27	Lymphoma, Large B-cell, Diffuse	treatment of diffuse large B-cell lymphoma	Withdrawn (2024)	1	Re-assessment of Orphan criteria at the time of type II variation: significant benefit not proven (OMAR, 2024).
EU/3/22/2634	2023	Conditional approval	AbbVie	epcoritamab	Tepkinly®	L01FX27	Lymphoma, Large B-cell, Diffuse	treatment of follicular lymphoma	Withdrawn (2024)	1	Re-assessment of Orphan criteria at the time of type II variation: significant benefit not proven (OMAR, 2024).
EU/3/03/139	2007	Standard	Janssen-Cilag	bosentan	Tracleer®	C02KX01	Systemic sclerosis	Treatment of systemic sclerosis (scleroderma)	Withdrawn (2014)	7	Pediatric data available. MAH preferred SPC extension (EP0526708).
EU/3/12/1080	2016	Conditional approval	AbbVie	venetoclax	Venclyxto®	L01XX52	Leukemia, Lymphocytic, Chronic, B-Cell	Treatment of chronic lymphocytic leukaemia	Withdrawn (2018)	2	Re-assessment of Orphan criteria at the time of type II variation: significant benefit not proven (OMAR, 2018).
EU/3/02/131	2005	Standard	UCB Pharma	sodium oxybate	Xyrem®	N07XX04	Narcolepsy	Treatment of narcolepsy	Withdrawn (2010)	5	Type II variation to add a new indication (treatment of moderate to severe symptoms of fibromyalgia). The new proposed indication does not fall within any orphan condition.

Concerning the 23 afore-mentioned OMPs, three possible motives for the premature removal of orphan designations can be identified:

1. A re-assessment of orphan designation criteria at the time of the granting of a change to the terms of the marketing authorization (extension of the current therapeutic indication): significant benefit of the product for the new proposed indication was not proven, as per Article 3(1)b of Regulation (EC) No 141/2000 (9 of 23; 39.1%);
2. The MAH prefers to apply for a 6-month extension of the supplementary protection certificate (SPC) instead of an extension of the market exclusivity period when the results of the Paediatric Investigation Plan became available (7 of 23; 30.4%);
3. A re-assessment of orphan designation criteria at the time of approving a change to the marketing authorization terms (to add a new therapeutic indication): The proposed new indication does not qualify for orphan designation, leading to the removal of the approved orphan designation(s) for that product, as per Article 7 of Regulation (EC) No 141/2000 (5 of 23; 21.7%).

For two products, we have not been able to directly determine the motive underpinning the (voluntary) removal of the OD status. In the case of Arzerra^®^, we believe the MAH removed the OD (in December 2018) as a preliminary step for the planned withdrawal of the MA, which happened a few months later (February 2019). However, this was not clearly mentioned on the EMA website, contrary to what is observed for the other OD withdrawals (*n* = 13) that occurred at the time of the MA withdrawal. For Adcetris^®^, the reason for the premature withdrawal is not clear. The MAH submitted a type II variation to extend the indication. However, due to a lack of data supporting the proposed indication, the CHMP raised major objections, which could not be solved during the “stop the clock” phase, and the MAH decided to withdraw its application [11]. Since the change to the indication was not approved, we would have expected no consequences for the OD status. However, 3 months after the withdrawal of the variation, the MAH removed the ODs of Adcetris^®^.

## Discussion

The procedure for obtaining orphan designation and subsequent removal from the Community Register of Orphan Medicinal Products is outlined in Article 5 of the Orphan Regulation (EC) No. 141/2000. The sponsor can submit the application to the Agency at any point during the development of the medicinal product. The COMP reviews the application and provides an opinion within 90 days. It is important to understand that receiving orphan designation from the EC does not automatically grant marketing authorization for the medicine. To market the product in the European Economic Area (EEA), a marketing authorization (MA) issued by the EC is necessary. If the applicant chooses to proceed with an application for a marketing authorization as an orphan medicinal product, a second evaluation of the orphan criteria by the COMP will occur alongside the assessment of the marketing authorization application carried out by the CHMP. This second review of the orphan criteria is required in order to maintain the orphan designation and qualify for the 10-year market exclusivity. In practice, this means that, in the EEA, no MA will be granted for a similar medicinal product with the same therapeutic indication for 10 years.

Among the 84 withdrawals that happened upon request of the sponsor, 30 (or 35.7%) occurred at the time of the MA granting. These 30 medicines were approved by the EC and simultaneously removed from the Community Register of Orphan Medicinal Products since they did not meet the orphan designation criteria. For these 30 medicines, the 10-year market exclusivity never applied.

Given that the orphan maintenance assessment reports (OMAR) are only available since 2018, we could only identify the motive for the OD withdrawal for 16 of these 30 products. For each of these 16 products, the COMP raised issues regarding the claim for significant benefit. In addition, for 6 products, the COMP indicated that the prevalence data required re-calculation.

One of the most critical and complex orphan designation criteria is the concept of ‘significant benefit’. Article 3.1(b) of the Orphan Regulation states that a medicinal product shall be designated as an orphan if the sponsor can prove that “there exists no satisfactory method of diagnosis, prevention or treatment of the condition in question that has been authorised in the Community or, if such method exists, that the medicinal product will be of significant benefit to those affected by that condition” [1]. When applying for OD, a set of plausible assumptions is often sufficient to claim “significant benefit” [12]. This means that evidence of improvement must be provided [13]. However, when the COMP reviews the OD criteria at the time of the MA granting, the evidence must come from clinical data, The applicant can claim that the product is of significant benefit if these data demonstrate a clinically relevant advantage (improved efficacy or improved safety profile) or if the product brings a major contribution to patient care (ease of use, due to a new mode of administration, or a dosing schedule significantly reducing the number of injections needed) [14]. This is one of the reasons why proving ‘significant benefit’ can be challenging at the time of granting the MA, leading to the loss of the OD at this stage.

In addition, the concept of ‘satisfactory methods’ is not always fully understood. In the orphan regulatory practice, ‘satisfactory methods’ refer to all products authorized in the EU for the orphan condition as proposed for designation. Satisfactory methods can, therefore, be identified with reference to Sect. 4.1 of the respective Summary of Product Characteristics (SmPC). Importantly, in orphan designation procedures, the set of products used to establish significant benefit comprises all medicines authorised to treat the said condition. For marketing authorization procedures, the final indication as agreed by CHMP and reflected in the new SmPC will determine which products are to be considered relevant for the significant benefit comparative exercise [13, 15].

With respect to this matter, we identified the case of treosulfan (Trecondi^®^), which was removed from the Community Register of Orphan Medicinal Products at the time of the granting of the MA due to a lack of clinical data supporting the clinical benefit compared to the satisfactory methods already authorised. At the time of the MA approval, the COMP performed the re-assessment of the orphan criteria and found that, as regards the existence of a significant benefit, the applicant had established that Trecondi^®^ provided a significant benefit in comparison with busulfan (Busilvex^®^) and thiotepa (Tepadina^®^), but not in comparison with melphalan- and cyclophosphamide-based medicinal products [16]. Consequently, the medicinal product Trecondi^®^ was removed from the Community Register of Orphan Medicinal Products. However, the applicant decided to appeal the decision in front of the General Court of the EU (GCEU), arguing that melphalan- and cyclophosphamide-based medicinal products should not be considered as ‘satisfactory methods’ since the approved therapeutic indications of these products are not the same as the indications covered by Trecondi^®^. The Court concluded that, indeed, melphalan- and cyclophosphamide-based medicinal products cannot be regarded as ‘satisfactory methods’ within the meaning of the first alternative of Article 3(1)(b) of Regulation No 141/2000 and, therefore, annulled article 5 of the European Commission’s Implementing Decision C (2019) 4858 (final) of 20 June 2019 [17]. Further to the Judgment of the GCEU of 23 September 2020, the Commission adopted an Implementing Decision on 24 November 2020, authorising the medicinal product as orphan [18].

After the MA has been granted to a specific product, and until its withdrawal (or revocation by the Health Authority), the MA enters what regulatory professionals call the “Maintenance phase”. During that phase, different regulatory activities are undertaken. These post-approval activities include variations (according to Regulation (EU) 2024/1701 of 11 March 2024 amending Regulation (EC) No 1234/2008 [19]), notifications (according to Article 61(3) of Directive 2001/83/EC [20, 21]), renewals (in accordance with Article 24 of Directive 2001/83/EC [20]), and MA transfers to another company [22]. Variations are changes to the terms of a marketing authorization and can be triggered by different reasons: new regulatory requirements (ex: submission of nitrosamines risk assessments), quality and/or manufacturing updates (ex: submission of a new active substance manufacturer), administrative change (ex: change in the address of the MAH), or safety updates (ex: change to the summary of product characteristics and/or package leaflet to implement the outcome of a procedure concerning periodic safety update reports or of a post-approval safety study). Variations can also be triggered for commercial reasons, for instance, when the MAH intends to add a new therapeutic indication or modify the approved one to increase the target population. These changes are classified, according to the current variation’s guideline [23], as type II variations. Type II variations are considered major changes to a marketing authorization that may have a significant impact on the quality, safety, or efficacy of a medicine. For this reason, they require assessment by the Health Authority and can only be implemented after formal approval has been issued by the EMA. Hence, along with the evaluation of the variation’s content, the COMP will conduct an additional assessment of the OD criteria if the change affects the therapeutic indication initially approved. Therefore, one of the reasons for the early removal of the OD is the lack of clinical data demonstrating a significant benefit for the updated/extended indication. In that case, the COMP recommends the removal of the product from the Community Register of Orphan Medicinal Products. We identified 9 out of 23 OMPs for which the OD had to be removed at the time of change to the terms of the marketing authorization (i.e., extension of indication).

An alternative motive for the removal of the orphan designation(s) is when the MAH requires the addition of a new therapeutic indication that does not fall within any orphan designation, as per Article 7 of Regulation (EC) No 141/2000 [1]. In the period studied, 5 out of 23 OMPs lost their OD due to the addition of a new therapeutic indication that was not rare. Therefore, the MAH had to request the removal of the OD since it is not possible to keep orphan and non-orphan indications in the same MA.

As previously mentioned, when an OMP receives an MA and the COMP recommends maintaining its OD status, that OMP will benefit from 10 years of market exclusivity (+ 2 years if paediatric). However, Article 8 (3) of Regulation (EC) No 141/2000 [1] describes three types of derogations from the market exclusivity provided under Article 8 (1) of that Regulation [24]. If an applicant applies for an MA for a similar product for the same indication, there are some exceptions that allow the Agency to recommend an MA for that similar product. One possibility is that this new product is of significant benefit (it is safer, more effective or otherwise clinically superior), the other possibility is the inability of the original MAH to supply the product in sufficient quantities (an event which was never mentioned in the EPARs we examined) and the other one is if the MAH of the first OMP gives his consent.

There is one example of consent given by the MAH to allow the authorisation of a similar product for the same indication. In 2001, Novartis obtained an MA for imatinib (Glivec^®^) to treat chronic myeloid leukaemia. In 2006, an orphan designation was granted to nilotinib (Tasigna^®^) for the same indication. One year later, Novartis applied for an MA for nilotinib. Since both products were considered similar and intended to treat the same orphan condition, nilotinib impinged on the market exclusivity of imatinib. Under the derogations foreseen in the Orphan Regulation, a company can give consent to a second applicant for a similar product entering the market. No controversy arose, since both products are from Novartis [25]. Had the two involved companies been competitors, obtaining such a consent would likely be difficult, albeit not impossible. It would depend on the type of agreement and the willingness to negotiate of both companies. In our opinion, it is highly probable that a financial/commercial transfer would be at the core of the agreement. Presumably, that would not be considered illegal or unethical, since it is foreseen in the Regulation. Information regarding the similarity assessment outcome and any derogations applicable can be found in the European Public Assessment Report (EPAR) of the respective product available on the EMA website. We did not identify any case of consent given by a company to its competitors.

The period of market protection is extended by two years when paediatric studies have been conducted. This protection against competing products can also be combined with intellectual property (IP) protection and data exclusivity, which prevents generic companies from relying on the original toxicological, pharmacological, and clinical data created to meet regulatory requirements.

With the implementation of the Paediatric Regulation (EC) No 1901/2006 [26], on 26 January 2007, the submission of a paediatric investigation plan (PIP) became mandatory for all marketing authorisation applications, irrespective of whether the medicine targets an orphan disease, under Article 8(3) of Directive 2001/83/EC (i.e., full applications). This requirement also applies when an MAH wants to add a new indication, pharmaceutical form or mode of administration for a medicine that is already authorised and covered by intellectual property rights. A PIP is a development plan aimed at ensuring that the necessary data are obtained through studies on child populations. These PIPs (deferrals, waivers or modifications) are assessed by the Paediatric Committee (PDCO). The PDCO formulates an opinion, and the Agency adopts a decision. Applicants must follow the agreed PIPs exactly [27], and once the plan is complete, the EMA checks that the company did comply with the agreed measures listed in each PIP. A successful compliance check (i.e., all studies completed with results implemented in the label) leads to a reward. The reward system envisages three scenarios [28]: (1) a 10-year market protection (including 8 years of data exclusivity) for paediatric-use marketing authorisations (PUMA), which are a dedicated MA developed exclusively for use in the paediatric population that are no longer covered by a supplementary protection certificate (SPC); (2) a 6-month extension to the SPC for new medicines or on-patent authorised medicines; (3) two additional years of market exclusivity in the case of orphan drugs.

Under the Paediatric Regulation, the orphan medicine sponsor cannot choose which incentives to benefit from (i.e. between a 6-month SPC extension or 2 additional years of marketing exclusivity). We identified 7 OMPs for which the OD has been removed when the PIP results became available. This suggests that, in some cases, the MAH preferred to benefit from a 6-month SPC extension for non-orphan use instead of the 2 additional years of market exclusivity applicable to OMPs. Therefore, the applicant requests the removal of the OD to receive an extension of the SPC.

Montanaro et al. [6]. conjectures that the reason for early removals could be the existence of commercial agreements between companies when a second applicant intends to apply for an MA with a similar indication, but it cannot prove a significant benefit. In that case, according to Montanaro et al.., the second applicant ought to have financially compensated the first MAH, in exchange for an early withdrawal. However, the available data do not support this conjecture.

Montanaro et al. discuss two examples. The MA of Cyramza^®^ (ramucirumab) in the treatment of gastric cancer was issued in December 2015. Exactly one year later, the MAH requests the removal of the OD. The reason is clear: the new indication (metastatic colorectal cancer) added through a type II variation, approved in 2015, does not fall within any orphan condition [29]. Therefore, the MAH had to request the withdrawal of the OD. The second example in Montanaro et al. concerns Lenvima^®^ (Lenvatinib; Esai), which was approved in 2015 for the treatment of papillary thyroid cancer and follicular thyroid cancer. In 2017, the MAH submitted a type II variation to add a new therapeutic indication (treatment of hepatocellular carcinoma). At the time of the re-assessment of the OD criteria, the COMP concluded that the MAH should recalculate the prevalence of the condition and that the claim of a clinically relevant advantage could not be supported since the main study submitted only demonstrated non-inferiority in the primary endpoint [30]. Therefore, the MAH had to request the withdrawal of all ODs granted to this product.

Second, remember that the original MAH does not have to withdraw its OD to let a competitor submit a marketing authorisation application. As discussed, consent between companies foreseen in the Legislation is, at least in theory, possible.

Third, removal of the OD by the first MAH could be commercially risky since it would allow other competitors to enter the market, as market exclusivity would no longer apply.

Fourth, for all cases of early removals identified (23 products), we have checked if, within a year of the OD removal, any other (similar) product has been approved. We did not identify *any* such case. Therefore, we conclude that there is no evidence that the motive of early removals of OD in the EU is a “pay to enter agreement”. The fact that most of the products for which the OD has been prematurely removed are oncological products is due to the fact that about 40% of all OMPs approved have oncological indications, as already reported by other authors [31–33]. From the results of our study, we observed that a significant percentage of these oncological orphan medicines have more than one therapeutic indication (examples: Glivec^®^, Revlimid^®^, Imbruvica^®^, Jakavi^®^, Lenvima^®^, Vyndaqel^®^, etc.). This means that type II variations have been submitted and, as a consequence, a re-assessment of the OD criteria took place. Thus, it is not strange that these early removals of ODs are more frequent in the group of oncological OMPs.

In summary, the assessment of the OD criteria for a determined medicine takes place at three different moments: at the time of the initial OD application (in the pre-authorisation phase), at the time of the MA granting and at the time of the extension of the therapeutic indication(s) (post-authorisation phase). This reassessment step does not exist in the United States. This may influence the choice of regulatory strategy for orphan drug designation and could explain the differences in the number of applications submitted to the FDA and the EMA. Between 1983 and 2019, a total of 5,099 medicines received orphan drug designation in the US. In the EU, about 2890 ODs have received a positive opinion between 2000 and 2023 [34–37]. It would be interesting to conduct the same study but analysing the OD status of the orphan drug products approved in the US since the implementation of the Orphan Drug Act in 1983 and compare the results (in terms of the shares and motives of OD withdrawals upon request of the sponsor).

Currently, in the EU, it is not mandatory to mention the reason behind a request to withdraw the OD of an OMP. If the motive is related to the re-assessment of the OD criteria at the time of a change to extend or add a new indication, this will be found in the OMARs available on the EMA website since 2018. In other cases (e.g. patent extension preferred instead of + 2 years of market exclusivity), the trigger for the premature removal is not traceable.

This study reinforces the need for more transparency regarding the reasons behind the withdrawals of OD upon request of the MAH. We consider the current ongoing discussion on the new General Pharmaceutical Legislation an opportune moment to revise the requirements and procedures for removal of OD. Moreover, the EMA is now implementing the IRIS platform, which is a portal for handling product-related scientific and regulatory procedures with EMA, including submission of applications for OD or removals of OD. It would be desirable for the EMA to require that the MAH indicate the motive for the early OD withdrawal in the IRIS platform. This motive could then be encoded on the EMA website under the section “More Information on < Product Name>”. Currently, under this section, only a general sentence is mentioned informing that the product was originally designated an orphan medicine for the indication X and the date of OD withdrawal. But the specific reason for the OD withdrawal request is not mentioned.

In April 2023, the EC proposed changes to the current Pharmaceutical Legislation, drafting one directive [38] and one regulation [39] which will replace the existing GPL (Regulation 726/2004 and Directive 2001/83/EC) and the legislation on medicines for children and for rare diseases (Regulation 1901/2006 and Regulation 141/2000/EC, respectively) [9]. In April 2024, the European Parliament (EP) made amendments to some of the proposed changes for both the Regulation [40] and Directive [41]. Following this, the EC’s position will be adopted, and negotiations between EC, EP and Member States have been launched in the summer of 2025. It still has to be followed by a second reading and final adoption. In principle, the main incentive for completing paediatric studies in compliance with a PIP will remain in the form of a 6-month SPC extension. The difference is that this 6-month SPC extension will also apply to orphan medicines, meaning that the separate incentive of a 2-year additional market exclusivity for paediatric indications will no longer be applicable. Being so, this proposal will benefit companies that no longer have to withdraw the orphan designation to take advantage of this incentive. According to Article 66 (4) of the proposed new Regulation, “an orphan designation ceases to be valid once an orphan medicine sponsor has obtained a marketing authorisation for the relevant medicinal product in accordance with Article 13(2)”. This OD will be valid for seven years. The applicant can, at any time, request the withdrawal of the orphan medicine [39]. It will be interesting to analyse the trends in OD removals upon the request of the sponsor, the reasons, and characteristics of these removed ODs after the implementation of the new revised Regulation. The European Parliament proposed, as an amendment to article 66, stating that “The orphan medicine sponsor *may* provide a reasoned justification for a withdrawal request, which shall be made publicly available”. However, no formal obligation is mentioned, neither by the European Commission nor the European Parliament, to indicate the reason for OD withdrawal. We believe it is important to create a Guidance document for applicants, issued by the Agency, which introduces this requirement for OD removals at the request of the sponsor. This would help the scientific community and regulators to better understand the development of orphan medicines and the associated challenges.

## Conclusion

In summary, we identified three main reasons for the early OD removals: a lack of clinical evidence supporting the significant benefit, a 6-month extension of the SPC when the MAH gets a positive compliance check of the PIP, and the new therapeutic indication added through a change to the terms of the marketing authorisation is not rare. Across all these early withdrawals, there is no evidence of commercial agreements between pharmaceutical companies (“pay to enter” agreements). The reason for requesting the withdrawal of the OD by the sponsor is not always clear and often requires the consultation of different documents available at the EMA website/European Patent Register to find out the possible reason. It is currently not mandatory for the MAH to mention the reason for the withdrawal request in the cover letter to the EMA. However, we consider it essential to keep the transparency of any regulatory action related to (orphan) medicines.

## Data Availability

We used public and freely available data from European public assessment reports (EPARs), orphan drug designation reports, orphan maintenance assessment reports (OMARs) from the web page of the European Medicines Agency (EMA) and information about the legal status of the patents and respective SPC retrieved from the European Patent Register. Please contact the author for specific data requests.
